# Beyond the Usual Suspects: Physiological Roles of the Arabidopsis Amidase Signature (AS) Superfamily Members in Plant Growth Processes and Stress Responses

**DOI:** 10.3390/biom11081207

**Published:** 2021-08-13

**Authors:** José Moya-Cuevas, Marta-Marina Pérez-Alonso, Paloma Ortiz-García, Stephan Pollmann

**Affiliations:** 1 Centro de Biotecnología y Genómica de Plantas, Universidad Politécnica de Madrid (UPM)—Instituto Nacional de Investigación y Tecnología Agraria y Alimentación (INIA), Campus de Montegancedo, Pozuelo de Alarcón, 28233 Madrid, Spain; marta.perez.alonso@slu.se (M.-M.P.-A.); p.ortiz@upm.es (P.O.-G.); 2 Umeå Plant Science Centre, Department of Forest Genetics and Plant Physiology, Swedish University of Agricultural Sciences, 901 83 Umeå, Sweden; 3 Departamento de Biotecnología—Biología Vegetal, Escuela Técnica Superior de Ingeniería Agronómica, Alimentaria y de Biosistemas, Universidad Politécnica de Madrid (UPM), 28040 Madrid, Spain

**Keywords:** arabidopsis, amidase signature superfamily, growth, stress, auxin, abscisic acid, amidase, indole-3-acetamide, indole-3-acetic acid, fatty acid amide hydrolase

## Abstract

The diversification of land plants largely relies on their ability to cope with constant environmental fluctuations, which negatively impact their reproductive fitness and trigger adaptive responses to biotic and abiotic stresses. In this limiting landscape, cumulative research attention has centred on deepening the roles of major phytohormones, mostly auxins, together with brassinosteroids, jasmonates, and abscisic acid, despite the signaling networks orchestrating the crosstalk among them are so far only poorly understood. Accordingly, this review focuses on the Arabidopsis Amidase Signature (AS) superfamily members, with the aim of highlighting the hitherto relatively underappreciated functions of AMIDASE1 (AMI1) and FATTY ACID AMIDE HYDROLASE (FAAH), as comparable coordinators of the growth-defense trade-off, by balancing auxin and ABA homeostasis through the conversion of their likely bioactive substrates, indole-3-acetamide and *N*-acylethanolamine.

## 1. Introduction

Beneath the apparent simplicity of the sessile lifestyle of plants, an intricate hormonebased machinery becomes crucial to face an often hostile environment. Infectious pathogens, herbivorous predators, soil salinity, drought, or temperature fluctuations, are among the diverse biotic and abiotic stresses challenging their survival and optimal reproduction [1,2]. In this restricting scenario, the sensing of these stimuli activates the concerted action of diverse interconnected signaling pathways, wherein the combinatorial action of few major phytohormones orchestrate a wide range of specific physiological processes, depending on both the responding tissue and the stimulus itself [3,4,5].

Under favorable circumstances, a vast miscellany of plant growth and developmental aspects, such as promotion of cell elongation, expansion, and differentiation, have been so far majorly ascribed to those signaling molecules of the auxin class [6,7,8]. In contrast, brassinosteroids, jasmonates (JAs) and abscisic acid (ABA) are widely known elicitors of stress responses to biotic and abiotic factors [9,10,11,12,13,14,15,16,17,18], in most cases, adapting the plant growth strategy by means of growth rate reduction and the anticipation of vegetative to reproductive phase transition [19,20,21]. However, the underlying crosstalk by which, e.g., JAs and ABA impact auxin homeostasis, thereby coordinating the growth-defense response trade-off, and thus rewiring transcriptional circuits to maximize phenotypic fitness for the prevailing stress condition, also remains largely elusive. Indeed, despite the cumulative knowledge on auxin homeostasis control, ranging from *de novo* biosynthesis to inactivation through conjugation, sequestration and degradation [22,23], the biosynthesis of the major auxin-representative indole-3-acetic acid (IAA) is still inconclusive [24,25].

Hence, this work provides updated information on the Arabidopsis AS superfamily members, focusing our main attention not only on the Amidase 1 (AMI1)-dependent IAA biosynthesis, but also on the hydrolysis of *N*-acylethanolamines (NAEs) by a fatty acid amide hydrolase (FAAH), based on the strong evidence to bridge their growth-inhibiting shared roles to ABA signaling pathway. Thereby, our review may fuel future synergistic research approaches, integrating the converging roles of their preferred substrates in early plant growth, thus leading future biotechnological innovations to sustainably increase crop yields and meet the worldwide growing demand.

## 2. The AS Superfamily

The ubiquitous AS members are a numerous group of amidohydrolases widely distributed throughout prokaryotes and eukaryotes (such as bacteria, mammals and plants). AS members are highly divergent both in terms of substrate preferences and function [26,27,28]. The products of their hydrolytic activity are the resulting carboxylic acids and either amine or ammonia compounds derived from the C-N amides bond. The shared feature of all the family members is this so-called amidase signature (AS), which refers to a conserved serine- and glycine-rich motif of 50–130 amino acids containing an unorthodox Ser-*cis*Ser-Lys catalytic triad, instead of the typical Ser-His-Asp triad found in the active sites of serine proteases [27,29,30].

This group of enzymes include: the plant AMI1, a specific indole-3-acetamide (IAM) amidohydrolase that synthesizes IAA from IAM [31]; FAAH, an integral membrane protein which hydrolyzes NAEs, thereby terminating their actions [27]; Glu-tRNAGln amidotransferase, an heterotrimeric enzyme required for the formation of appropiately charged glutamine codons during translation [32]; allophanate hydrolase, crucial for urea usage as a nitrogen source by diverse organisms, by means of allophanate to ammonium and carbon dioxide conversion [33]; peptide amidase (PAM), for selective hydrolysis of the C-terminal amide bond of peptides [34]; the bacterial malonamidase E2 (MAE2) catalyzing the hydrolysis of malonamate to malonate and ammonia, by symbiont bacteroids for transport of fixed nitrogen to plant cells [35].

### 2.1. The Arabidopsis AS Superfamily Members

Only two proteins, AMI1 and FAAH, out of the seven different coding genes constituting this small enzyme family were characterized for their enzymatic activity [27,30,31,36,37,38]. A third isoform (At5g09420) is seemingly located in the outer mitochondrial membrane as part of the preprotein translocon (Tom-complex) [39], whereas the fourth one (At3g17970) associates by protein cross-linking with those of the outer envelope of chloroplast Toc-complex [40,41], and both most probably lack enzymatic activity. The remaining three members are yet to be functionally characterized, although At4g34880 gene might function in leaf vascular tissues during sink-to-source transition [42], and At3g25660 likely interacts with the Glu-tRNA(Gln) amidotransferase subunit B (GAT-B) [43].

#### 2.1.1. AMI1

##### The Atypical Member of the Family

**Figure 1:**
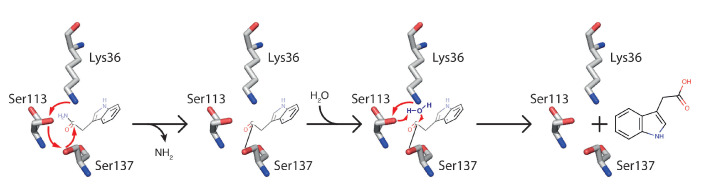
Proposed mechanism for the conversion of IAM by Arabidopsis AMI1. The enzymatic reaction initiates with a nucleophilic attack of the α carbon atom of IAM by Ser137. Lys36 acts as a catalytic base and, possibly, receives protons from Ser113 in a proton relay. After the liberation of ammonia, an intermediate acyl-enzyme complex is formed, which disintegrates after the addition of H_2_O and the release of the reaction product, IAA.

The apparent molecular mass of AMI1 is around 45 kDa, its subcellular localization is in the cytoplasm, and the canonical residue composition of the Ser-*cis*Ser-Lys triad remains conserved (PS00571 in the PROSITE dictionary) [36,44]. However, the C$X_{3}$C motif is missing (this additional pattern is only conserved in a restricted number of AS members, enabling nitrile cleavage capability), thus excluding the accessory Cys-*cis*Ser-Lys catalytic center described in *Rhodococcus rhodochrous* strain J1 or *Sulfolobus solfataricus* [45]. Functional and structural comparative analyses were performed taking advantage of three-dimensional homology-based protein models, revealing conspicuous similarities between AMI1 and rat FAAH regarding the arrangement of the active-site residues, and explaining the dramatic impact of several AMI1 functional mutations on its enzymatic activity linked to the essential Ser137 residue. Over the past years, two different mechanisms for the enzymatic conversion of primary and secondary amides, respectively, have been proposed for AS enzymes. Concerning the initially proposed mechanism [46], Ser137 acts as a nucleophile, while Lys36 is a proposed catalytic base and proton acceptor from Ser113 of AMI1, which likely collaborate in a proton relay system. As depicted in Figure 1, the conversion of IAM includes the formation of an acyl-enzyme intermediate stage. However, detailed information is still missing, and further investigation is needed. On the other hand, the alternative mechanism proposed by Labahn and colleagues (2002) [47] assumes a protonated lysine residue and a tetrahedral transition state over the course of the enzymatic conversion. However, given a pK${}_{R}$ of 10.53 for the lysine residue and an only marginally basic environment in the cellular setting, it seems as if the initial mechanism must be favoured for the activity of AMI1. Besides, AMI1 activity is drastically impaired by very low concentrations of phenylmethanesulfonyl fluoride, as is the case for most of the AS members, thus underscoring the catalytically active serine residue in the center of its polypeptide active site [26].

Aside from the expected common features to other AS members, AMI1 exhibits some striking differences, being the only member with indole-3-acetamide hydrolase activity, with IAM and phenylacetamide as its preferred substrate [36]. This amidohydrolase shows minor reactivity towards oleamide and NAEs [48], as well as strong co-localized expression in tissues with high auxin content, thus suggesting a role of AMI1 in auxin biosynthesis [44]. In this line, AMI1 also converts 1-naphthaleneacetamide (NAM), a synthetic structural homologue of IAM, to 1-naphthaleneacetic acid (NAA), which is a strong auxin [49], similarly to *Agrobacterium tumefaciens IaaH* gene product acts against IAM and NAM [50]. On the other hand, significant evidences point to dimerization properties of AS hydrolases, such as MAE and FAAH, wherein a fragment of the N-terminal end was the selected locus for protein-protein interaction and membrane association experiments. However, both PAM and AMI1 show a monomeric mechanism of enzymatic action, as evidenced by blue native gel electrophoresis [51], yeast two-hybrid and bimolecular fluorescence complementation results, which allowed to distinguish between AMI1 and both MAE and FAAH [26]. Furthermore, unlike the characteristic bifunctionality reported for most AS family members, AMI1 lacks esterase ⁄ peptidase activities, and so, is unable to attack ester- or nitrile-bearing compounds at specific enzyme/substrate ratios [29,35,45,46]. Thus, AMI1 is incapable of converting IAA glucosyl ester or IAA methyl ester, nor N-substituted amides, such as IAA–amino acid conjugates and the characterized FAAHs substrates, NAEs [38].

##### Alternative Roads to IAA Biosynthesis

**Figure 2:**
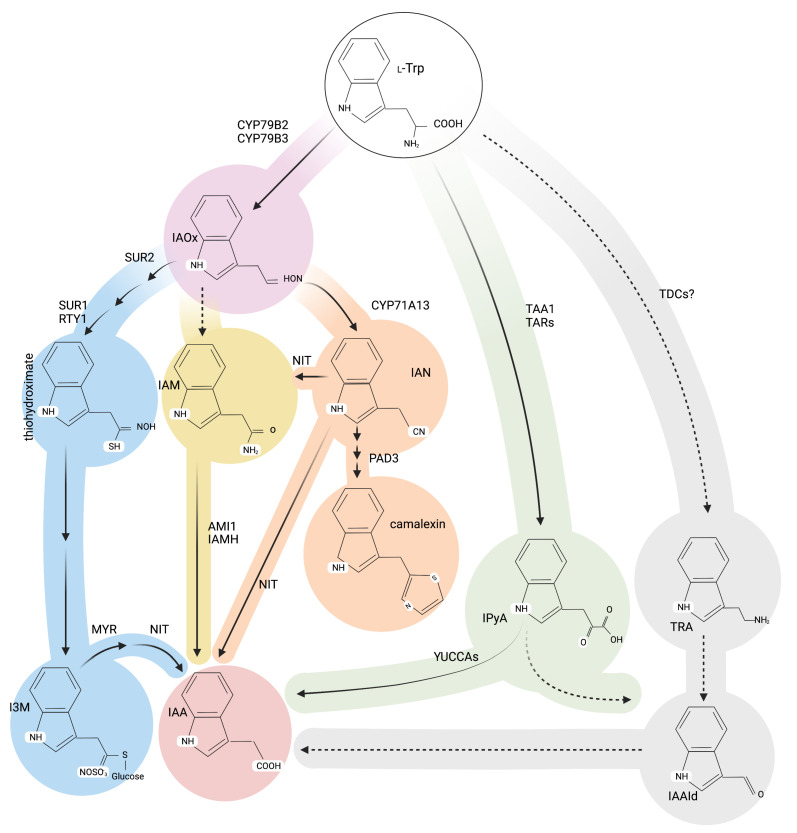
Abbreviated representation of *L*-Trp derived anabolic pathways for indole glucosinolate, camalexin, and indole-3-acetic acid biosynthesis in Arabidopsis. The reaction steps catalyzed by so far unidentified genes/enzymes are represented by dashed lines. Each postulated *L*-Trp derived shunts are coloured as follow: thiohydroximate in blue, IAM in yellow, IAN in orange, IPyA in green, TRA in grey. AMI1, AMIDASE1; CYP71A13, CYTOCHROME P450 MONOOXYGENASE 71A13, CYP79B2, CYTOCHROME P450 MONOOXYGENASE 79B2, CYP79B3, CYTOCHROME P450 MONOOXYGENASE 79B3, I3M, glucobrassicin; IAA, indole-3-acetic acid; IAAld, indole-3-acetaldehyde; IAM, indole-3-acetamide; IAMH, IAM HYDROLASE1-2, IAN, indole-3-acetonitrile; IAOx, indole-3-acetaldoxime; L-Trp, L-tryptophan; MYR, MYROSINASE; NIT, NITRILASE1-3; PAD3, PHYTOALEXIN DEFICIENT3 (CYP71B15); RTY, ROOTY; SUR1, SUPERROOT1; SUR2, SUPERROOT2 (CYP83B1),TAA1, TRYPTOPHAN AMINOTRANSFERASE OF ARABIDOPSIS 1; TAR, TRYPTOPHAN AMINOTRANSFERASE RELATED; TDC, TRYPTOPHAN DECARBOXYLASE; TRA, tryptamine.

General auxin biosynthesis is mainly derived from the indole-3-pyruvate (IPyA) anabolic pathway, wherein, the fine-tuning of tryptophan aminotransferases (TAA1/TAR2) and flavin containing monooxygenases (YUC1-11), becomes crucial to converting *L*-tryptophan (*L*-Trp) into IAA via the intermediate IPyA [52,53]. Either redundantly or in a parallel way with this major IPyA-derived auxin source, a reduced number of additional pathways are proposed to operate in higher plants [54,55,56,57], as shown in Figure 2. Among these routes, the IAM pathway was originally circumscribed to plant pathogenic bacteria and later proposed to operate in plants, based on three pivotal findings. Firstly, the IAM endogenous contents in *Prunus jamasakura*, *Citrus unshiu*, *Cucurbita maxima* and *A. thaliana* [51,58,59,60,61]. Secondly, the reported IAM amidase activity in *Triticum aestivum* and *Pisum sativum* tissues [62], *Oryza sativa* [63,64] and *Poncirus trifoliata* [65]. Lastly, and primarily based on in vitro and in vivo results, the AMI1 competence to convert IAM into IAA [26,36,37,66,67].

Within the Brassicaceae family and concretely in *A. thaliana*, up to 95% of IAM has been proven to originate from the precursor IAOx by a hitherto unidentified enzyme [68]. Moreover, IAOx is well-known as a significant metabolic bifurcation node, by which primary and secondary metabolism gets connected [69,70]. Thereby, IAOx stands out as the joint biochemical input required for the *L*-Trp-derived production of key glucosinolates [71], such as the Arabidopsis defensive compounds brassicin and camalexin [72], thus involving the transcription factors MYB34, MYB51, MYB122 and WRKY33 to promote glucosinolate and camalexin biosynthesis, respectively. [73,74]. Strikingly, although there are no indications of other IAM hydrolases intervening in the conversion of IAM into IAA within the AS family members [26,44], the aforementioned activity was not suppressed in loss-of-function mutants, thus inferring the existence of putative contributing enzymes outside this family [37]. In line with this assumption, and most probably explaining the remaining enzymatic activity, two recently reported formamidase-like proteins, IAMH1 and IAMH2, have been associated with this conversion [75,76].

However, despite the severely reduced IAM contents of *cyp79b2 cyp79b3* plants [68], both IAOx and AMI1 impaired mutants, exhibited minor IAA altered levels under standard conditions [31]. Herein, excluding the upregulation of *YUC8* and *ILL5/IAR3*, no other significant differentially expressed auxin homeostasis-related genes were identified in *ami1-2* [37]. Both the induction of *YUC8*, which takes part in auxin biosynthesis [13], along with the two comparable specific IAA-Leu and IAA-Phe IAA-amino acid hydrolases [77,78], might correspond to counteracting the lack of AMI1 activity. Conversely, inducible mutants overexpressing AMI1 (AMI1ind-2), showed significant overexpression of the auxin conjugation-related genes *UGT75D1* and *GH3.17*, together with a number of auxin transport- and signaling-related genes, including *LAX2*, *PIN4*, *PIN5*, *IAA1*, *IAA12*, *IAA14*, *ARF6*, *ARF7*, and *ARF16*, most likely in response to IAA overproduction. Further, no other alternative auxin biosynthesis routes, such as IAMH1 and IAMH2, were transcriptionally induced in *cyp79b2 cyp79b3* to balance the loss of IAOx source [68,69,79] or AMI1 activity [37], but demonstrated a significantly increased susceptibility towards pathogens [73]. Taken together, and despite the required AMI1 activity for appropriate culmination of concrete developmental processes, such as lateral root growth or seed maturation [37], all these evidences contrast with the role of AMI1 in overall auxin influx in Arabidopsis. Indeed, the fact that IAM has been found in several non-Brassica plant species commonly lacking the IAOx pathway [80], nourishes the assumption that a still unidentified tryptophan 2-monooxygenase, such the ones known from bacteria, i.e., iaaM and tms1 [81,82], are most probably leading to IAM by an alternative biosynthetic pathway.

##### The More IAM, the Less Plant Growth

AMI1 expression is majorly located in proliferating tissues, such as young seedlings and developing flowers, but is repressed during early stages of germination [37,44,51,83]. AMI1 is transcriptionally induced by its putative substrate, IAM, and, to a lesser degree, repressed by its reaction product, IAA [80]. Furthermore, in-depth mutant analysis provided further evidences to confirm the IAA formation from IAM *in planta*, most probably impacting cellular auxin homeostasis by means of balancing the IAM pool [37,84]. In this way, hindered AMI1 activity led to a moderate reduction of IAA contents (15 to 30%), but significantly increased IAM levels. Remarkably, this IAM accumulation exerted a negative impact on seed maturation, since both seed and embryo size were notably smaller [37]. These phenotypes were in accordance with the previously reported role of IAM as a transcriptional repressor of the elongation growth contributing K${}^{+}$ transporters *HAK/KT12* and *KUP4* [84,85]. Besides, the *ami1* mutants displayed a slight growth reduction of aerial parts, together with a significant reduction of root branching, as well as total root length and area [37]. On the contrary, conditional AMI1 overexpression produced phenotypes reminiscent of an auxin overproduction, as evidenced by growth retardation, curly leaf morphology and flowering anticipation [13,37,86,87]. It is noteworthy that, as a result of increased auxin levels, this downstream effects in AMI1 overexpressing mutants are probably neutralized by expression enhancement of a limited number of auxin conjugation-related genes, which, in turn, leads to a deactivation of the physiologically active free IAA [37].

Therefore, in the line of assessing the described plant growth repressing role of IAM, it was highly relevant to characterize the impact of endogenous IAM accumulation, aiming at deepening on the molecular and physiological mechanisms for IAM signal perception and integration. As a starting point to tackle this question, the consequences derived from the simultaneous genetic interruption of the indole glucosinolate and IAM pathways in *A.thaliana* were recently reported [31]. Strikingly, it was found that, normally germinating homozygous *ami1 rty* parentals, set a reduced number of siliques producing non-viable seeds. The offspring aborted germination just after radicle extrusion [31]. Consistently, it had to be concluded that impaired nutrient acquisition during seed filling was most probably inherited from heterozygous *rty* ancestors, thus explaining the parentals sterility.

To further dissect the observed IAM dwarfish effect on *ami1 rty* embryos and seeds, additional IAM and IAA mass spectrometric analysis of these homozygous seeds were performed, finding higher IAM:IAA ratios in *ami1 rty* relative to those of *wt* [31] and the *rty* allelic *sur1-1* mutant [68]. Alternatively, RNAseq transcriptional profiling of these double mutant seeds, found not only any differentially expressed genes involved neither in auxin metabolism nor camalexin biosynthesis pathway, but also, the induction of *WRKY33*, a transcriptional repressor of this camalexin anabolic route [31]. So, the initially hypothesized IAOx or IAM metabolic redirection into this pathway had to be finally discarded. On the contrary, the transcriptomics analysis provided evidence of significant repression of plant growth regulating processes in response to IAM treatment, for instance, hindering the expression of the growth-regulating factors *GRF3* and *GRF5* on IAM treated *wt* Arabidopsis seedlings [31]. Further, the identified downregulation of the TCP family members, *TCP10* and *TCP23* [31], underpins the observed AMI1-related growth and time flowering alterations, considering the key roles of these transcription factors in the control of shoot morphogenesis and developmental transitions [88,89].Of special note was also the downregulation of translation-related genes, involved primarily in ribosome biogenesis and assembly, as well as rRNA processing. Additionally, taking into account the impaired expression of carbohydrate metabolism- and amino acid biosynthesis-related genes, it has been proposed that IAM increased accumulation during seed development, impedes a proper remobilization of sugars and nitrogen-containing compounds from maternal tissues, which ultimately leads to developmental alterations responsible for nonviable seed production [31].

##### The AMI1 Connection: IAM-ABA Crosstalk in Stress Responses

**Figure 3:**
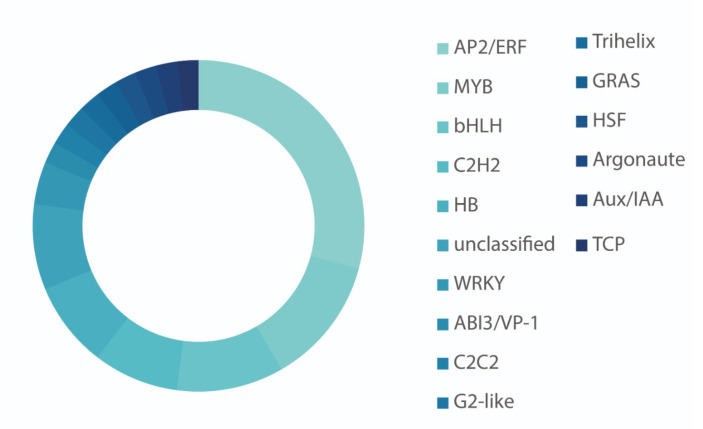
Differentially expressed transcription factor classes in *ami1-2* mutants according to Pérez et al. (2021) [37], based on an adjusted *p*-value of <0.05 and a fold-change of ≥1.5.

Altogether, IAM or other putative by-products, might act as a signaling molecule with prominent impact on gene expression regulatory processes. More than 12% of the differentially expressed IAM-responsive genes in *ami1-2* mutants belong to different transcription factor classes [37]. As represented in Figure 3, nearly 30% of these molecular components belong to the AP2/ERF, while 13 and 4% are MYB and WRKY transcription factors, respectively. The specific control of hormone and abiotic stress responses by AP2/ERF transcription factor networks, have been well-established [90]. In this context, recent transcriptomics approaches suggest a tight connection of IAM accumulation with biotic and abiotic stress responses, involving, e.g., key enzymes for JA and ABA production [31,37]. For instance, besides the already mentioned role of *YUC8* and *ILL5/IAR3* in auxin homeostasis, these *ami1-2* misregulated genes have been associated with biotic stress responses [13,91]. On another note, despite the negligible impact of salinity on AMI1 expression, osmotic stress conditions have been demonstrated to severely repress AMI1 transcriptional activity [37]. Thus, on the basis of the exhibited hypersensitivity of *ami1* seedlings in response to osmotic stress conditions, the repression of AMI1 activity has been proposed as a first line adaptation mechanism. Additionally, the remarkable number of identified differentially expressed small heat shock proteins by whole-genome transcript sequencing of *ami1 rty* seeds, points towards the misregulation of desiccation tolerance processes, involved in drought stress adaptive responses [31].

The above-mentioned osmotic stress responses involve ABA-dependent and ABA-independent pathways [92,93]. In the same way, ABA and gibberellins are indispensable determinants of seed development and dormancy [94]. In this context, it is particularly noteworthy the IAM connection with gibberellin signaling through the repression of *GNC* and *CGA1* transcription factors, both DELLAs downstream effectors [31]. Furthermore, the recently reported direct role of auxin in seed dormancy [95], as well as the transcriptional and metabolic crosstalk between IAA and ABA in seed development and germination [96,97], have led to propose an additional crosstalk connecting AMI1/IAM contents and ABA-related processes, with prosperous seedling development and germination. As demonstrated by the Arabidopsis germination rate reduction in response to IAM application, deficient AMI1 activity most probably enhances ABA production, and later results in aberrant embryo and seed size [37]. In the same manner, both differentially ABA-dependent pathways, have been shown to be activated through the transcriptional induction of the ABA synthesis gene, *NCED3*, either by means of IAM exogenous application, or as a result of ABA accumulation in the *ami1* alleles [37]. Hence, the osmotic stressed-induced transcriptional repression of AMI1, along with the resulting IAM accumulation, orchestrate the fine-tuning of ABA-dependent stress responses through NCED3-mediated ABA biosynthesis in Arabidopsis. However, no regulatory effect has been detected in a ABA-controlled AMI1 feedback loop.

#### 2.1.2. FAAH

##### What Lies Beneath the Structure

To date, FAAH is the sole known integral membrane protein within the AS enzymes, which catalyzes the hydrolytic central step in NAEs metabolism, converting these lipid signaling molecules into their corresponding free fatty acid and ethanolamine products, thus terminating their regulatory actions [29]. There are FAAHs representatives across diverse multicellular eukaryotes, including animals and plants [98], which feature key structural differences that account both for the types of bioactive NAEs, as well as for the substrate specificity promiscuity of those of mammals and plants [27,99]. Concretely in plants, this residue evolutionary adaptation have provided FAAHs with wide versatility, in terms of shaping physicochemically diverse catalytic cavities to accommodate both unsubstituted and oxygenated NAEs as signaling substrates.

Thus, the elucidation of the FAAH crystal structure [27], jointly with recent comprehensive in silico analysis of FAAH amino acid sequences in angiosperms, allowed to identify the conserved substitutions, located in no other than key residues around the acyl-binding pocket and the cytosolic access channel, responsible for the conformational variations distinguishing two separated groups: the Arabidopsis including FAAH (AtFAAH) group I, and the FAAH-like enzymes group II [30]. Interestingly, in contrast to the rest of dicot and monocotyledonous species explored, including the proposed common ancestor of all flowering plants, *Amborella trichopoda* [100], the phylogenetic analysis highlighted the Brassicaceae plant family (e.g., *A. thaliana*, *Brassica napus*, *Camelina sativa*) and castor (*Ricinus communis*), as those having FAAHs uniquely belonging to the group I. So, it has been suggested, in the basis of the joint presence of both FAAH groups in the *A. trichopoda* genome, an ancestral plant FAAH evolutionary bifurcation, predating angiosperms emergence, and later differential taxa-depending loss of orthologs. Consequently, throughout evolution, *A. thaliana* and its relatives had entirely lost the group II FAAH orthologs, whereas, e.g., group I is preferentially represented in Solanaceous species, while Gossypium or the leguminous plants have mostly group II instead of group I FAAHs [30].

Herein, homology modeling between *Glicine max* (soybean) group I and group II FAAHs were performed on the basis of the AtFAAH 3D structure template (PDB: 6DHV [27]). Thus, despite the retention of the distinctive catalytic triad (Ser-*cis*Ser-Lys) of the AS superfamily, this exhaustive inspection uncovered significant contrasts between the novel group II of FAAHs and the AtFAAH. In brief, conversely to the group I polar residues conforming the surroundings of the substrate-binding pocket, those of the group II are predominantly nonpolar and more reminiscent of mammalian FAAH, including some bulkier aromatic residues in the ligand-binding site. In the same manner, this lower group II hydrophilicity has been also predicted for its cytosolic access channel building residues, most probably to fit more hydrophobic head group substrates. Therefore, this structural diversity expands the so far underestimated plant signaling communication system, as evidenced by the putative vast collection of naturally occurring potential substrates for group II FAAHs, beyond NAEs themselves. So, according to emerging literature, these unnoticed signaling molecules may range from plant fatty acid amides, such as the alkamides [101], to microbial origin N-acyl *L*-homoserine lactones (AHLs), which are essential in N-acyl amide-mediated plant-microbe interactions [102].

##### One FAAH to Terminate Them All

Although there are marked structural differences between NAEs, the inherent FAAH promiscuity to hydrolyze these ubiquitous signaling bioactive acylamides, appear to be conserved throughout eukaryotic organisms [98,103], although it has been majorly investigated in vertebrates. So, these studies revealed the minor membrane phospholipid *N*-acylphosphatidylethanolamine (NAPE)-derived origin of NAEs, and further identified the mammalian phospholipase D (PLD) as the specific enzyme catalyzing the conversion of NAPE to NAE in vivo [104]. In plants, PLD-β and -γ isoforms are competent in vitro NAE converters [105], likely with additional participation of tissue-specific phospholipase A [106] or phospholipase C [107] mediated pathways. Thus, the resulting NAEs differ both in the acyl chain length (X = number of C atoms), and in the degrees of unsaturation (Y = number of double bonds), typically designated NAE X:Y, e.g., in the case of anandamide (NAE 20:4), the central bioactive molecule of the endocannabinoid signaling pathway in mammals.

However, the orchestration of numerous behavioral and physiological processes in vertebrates, not only depends on the anandamide binding to the membrane G protein-coupled cannabinoid receptors, CB1 and CB2 [108,109], but also on the mammalian FAAH ability to hydrolyze higher occurring CB receptor inactive NAEs, with the resulting competing pool of substrates available to FAAH [110]. On the other hand, in higher plants, anandamide is primarily absent, whereas NAE types range from 12 to 18C, the latter of which are the most abundant, and display zero to three double bonds [111,112]. Remarkably, oxylipin metabolites derived from polyunsaturated NAEs, as is the case with hydro(pero)xy derivatives of NAE 18:2 and NAE 18:3, are the main regulators on plant development, rather than their unsubstituted parent structures [113,114]. In addition, *A. thaliana* FAAH is equally efficient hydrolyzing either the hydroxylated or the unsubstituted NAE 18:2 [27], although the latter is the endogenous predominant derivative. So, both in animals and plants, the so-called “entourage effect”, wherein the resulting physiological effects depend on different competing NAE derivatives [115,116], is the evident operating signaling mechanism, which is ultimately terminated by FAAH action [29,98].

##### NAE Signaling Alterations: Plant Physiological Processes in Jeopardy

As in the case of animals, mounting experimental evidence has so far underpinned the crucial role of NAE signaling in regulating multiple plant physiological processes [117]. In the same way, the FAAH-dependent NAE signaling terminating role, has been proposed as the common catabolic mechanism operating both in mammals and plants [112]. Consistent with the elevated contents of NAEs in desiccated seeds [111], as well as their later depletion as imbibition and germination occur [48,118,119], the growth inhibiting properties of NAE derivatives have been extensively demonstrated.

Specifically, micromolar concentrations of exogenously applied NAE 12:0 and NAE 18:2, not only exert a dose-dependent reduction in seedling growth, but also provoke root cell and cytoskeletal organization alterations [119,120,121]. As expected, these triggered growth inhibitory effects were significantly attenuated in *AtFAAH* overexpressing seedlings compared to *wt*, with concordant reduction of endogenous NAE levels, and in contrast to loss-of-function *AtFAAH* lines, which exhibited enhanced sensitivity to NAE [119,122,123,124]. So, the resulting increased NAE hydrolytic activity by means of FAAH overexpression, was translated into enhanced overall seedling growth and early flowering [48,119,124], whereas *AtFAAH* knockouts did not display any other phenotype than the increased sensitivity to exogenous NAE [119]. Nevertheless, the counterpart to this increased growth of *AtFAAH* overexpressors was the jeopardized innate immunity to usually non-pathogenic organisms [125]. On the other hand, the early flowering *AtFAAH* overexpressing mutants displayed an associated induction of the flowering master regulator FLOWERING LOCUS (*FT*), under both inductive long day (LD) and non-inductive short day (SD) conditions [124,126,127,128,129]. Concretely, up to 30% content reductions in the specific derivatives NAE 12:0 and NAE 18:2 have been reported in *AtFAAH* overexpressors under 14 SD conditions growth, comprising a 9% less total NAE than *wt*. Moreover, *wt* Arabidopsis plants exogenously treated with NAE 12:0 showed a significant delayed flowering [124].

##### Convergent and Bifurcating Pathways at the NAE-ABA Signaling Crossroads

Over the past years, primarily based on *Arabidopsis* genetic research with ABA biosynthesis and ABA-insensitive (ABI) mutants, the negative regulating role of the ABA signaling cascade, inhibiting seed germination and arresting seedling growth, have been robustly established [130,131,132,133,134,135,136]. Thus, ABA triggers a myriad of instantaneous cellular responses [50,137,138] and gene expression changes [132,133,139], which include, e.g., the channel-mediated release of calcium and potassium, increased reactive oxygen species, nitric oxide release, sphingolipids and Glu receptors [137,138,140,141], as well as the activation of numerous genes by ABA-responsive elements (ABREs) [142]. Within the latter group, it is remarkable that some of these components have proven to be targets of NAEs, being the case, e.g., of certain ABA-mediated effects on seed germination physiology by binding to heterotrimeric G proteins [143,144,145,146], or the PLD-conversion derived by-product, phosphatidic acid (PA), involved in the regulation of ABA responses in guard cells [147,148,149] and seed germination [150]. Indeed, jointly with the increased growth of *AtFAAH* overexpressors, these lines display an enhanced sensitivity to ABA, likely due to elevated PA levels [122,123]. Notably, the effect of low concentrations of NAE in the nanomolar range were proven to effectively inhibit the in vitro PLD-α activity in a non-competitive fashion. In fact, later in vivo experiments showed that, specially, NAE short-chain saturated species, such as NAE 12:0, were the strongest PLD-a inhibitors [151].

Furthermore, even more compelling experimental results point towards the crosstalk between NAE and ABA signaling pathways to negatively regulate early seedling development [117,122,123]. In this line, either desiccation stress or exogenous ABA treatments arrest early seedling development, as evidenced by plants showing a significant root length reduction and smaller general seedling size, largely reminiscent to those of NAE supplied lines [120,121,122,130,131,133]. So, as expected, the combined treatment of NAE 12:0 and ABA exerts a synergistic effect on seedling growth arrest, for which it is essential a functionally unaltered ABA signaling pathway (involving ABI1, ABI2, ABI3, and ABI5), as well as associated upregulation of usual ABA-responsive genes [122,123]. Moreover, the levels of both growth-repressing metabolites, initially elevated in desiccated seeds, are gradually depleted over the course of germination and later during seedling development, following similar time course dynamics [118,119,152,153]. Besides, in the same manner, the tissues sensitivity to either ABA or NAE is gradually reduced as seedling growth progresses, concomitantly with upregulation of ABI3 transcripts. However, a higher level of complexity has been proposed to operate in controlling seedling development, since NAE can modulate the expression of genes other than those of the ABA-responsive cluster. In addition, since plants with an impaired ABA signaling pathway, as in the case of *abi3-1* seedlings, displayed arrested seedling growth in response to higher NAE 12:0 concentrations, but not to supplied ABA, it has been postulated that alternative ABA-independent mechanisms operate in regulating seedling development [122,123]. For instance, a clear evidence thereof, is either the ABA- or NAE-induced expression of the *RD29* drought response gene in absence of its major activator, the ABI3 transcription factor, within a narrow developmental window. Remarkably, even without growth inhibition effects, and outside this sensitivity time frame, there was only an ABA-responsive upregulation of *RD29B*, but not to NAE treatment, thus reinforcing the divergences between both metabolites in the regulation of growth [123].

## 3. Concluding Remarks and Future Perspectives

**Figure 4:**
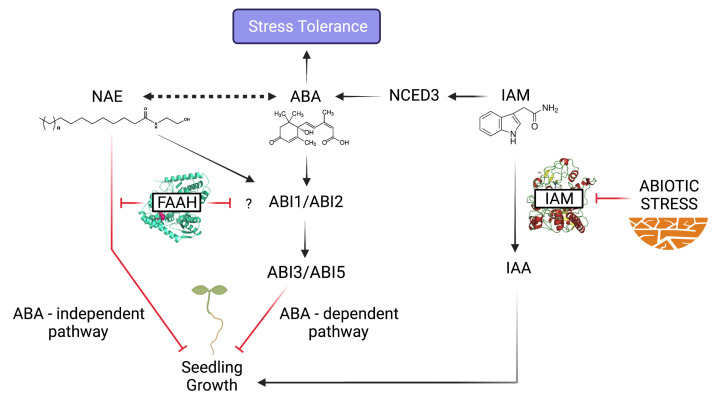
Schematic model integrating the converging growth inhibiting roles derived from NAE and IAM accumulated levels to trigger proper stress tolerance responses through ABA-dependent and -independent pathways.

In the basis of the recapitulated literature, it becomes clear that, both NAE and IAM signaling metabolites, can negatively impact plant growth processes, such as germination or early seedling development, either through the regulation of ABA-responsive or -non responsive genes. Thus, their common growth inhibiting roles, derived from elevated contents of either NAE or IAM, drive proper adaptive responses by means of modulating the growth-defense trade-off, as schematized in Figure 4. However, despite the remarkable differences between both amidases, majorly concerning to their source substrates [44], the AMI1 main role has been proposed as largely comparable to that of FAAH, regarding the interruption of IAM action by its catalysis to free IAA and NH${}_{4}$${}^{+}$ [37]. Thereby, unveiling the molecular basis and transcriptional networks involved in the integration of the IAM signal, by which the AMI1-dependent shunt connects auxin-regulated growth processes with plant adaptive responses to stress, will be comprehensively addressed in the short term. In the same way, it will be compelling to unravel the so far unknown mechanism of NAE perception, e.g., by means of particular receptor binding, or through both regulation of PLDa1 and PA levels. Indeed, beyond the reviewed FAAH role in seedling development, future experimental efforts will shed light on the FAAH-substrate platform as a plant-microbiota communication system, or serving as an alternative floral transition pathway by direct or indirect FT interaction. In this manner, all the data integrated herein mark a thrilling way towards a more productive agriculture, based on the potential use of overexpressing or knockout AMI1 and/or FAAH mutants, either to increase the productivity, or the resistance of these plants to adverse environmental conditions, respectively.
